# Supplementary material for: Pathophysiological Significance of Neutrophilic Transfer RNA-Derived Small RNAs in Asymptomatic Moyamoya Disease
Source: Cells. 2021 May 1;10(5):1086. doi: 10.3390/cells10051086 (PMC8147334; doi:10.3390/cells10051086)

2D Structure	Local AU	Position

```
592                              8mer-1a   625
5'-ccaacaGACAAACATCTAAAGTAAAGACGGAAAa-3' utr
         || || || ||| | |    |||||||
3'-uuugcaCUAUUGGU-GAUGUGA----UGCCUUUg-5' tRF
             16 15141 3       7 6 5 4 3 2
              3' Pairing         Seed
```

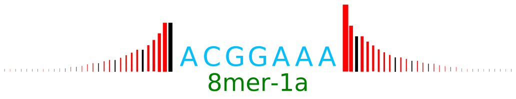

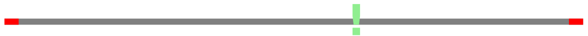

2D Structure	Local AU	Position

```
499                          8mer-1a  527
5'-tgtCCTG-TGCCTAGAAGTGTCACGGAAAa-3' utr
      | || |: |:|  | ::: |||||||
3'-uuuGCACUAUUGGUGAU-GUGAUGCCUUUg-5' tRF
                16151413   7 6 5 4 3 2
                3' Pairing    Seed
```

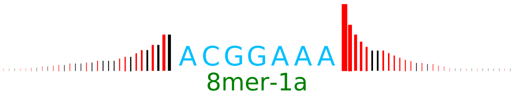

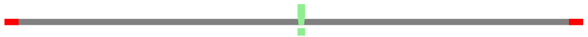

Supplement: Supplementary file 1 [file cells-10-01086-s001.zip › cells-1149161 3rd supplementary materials/Data Supplements/Data Supplement V.zip!/Data supplement 6-mRNAs targets of tsRNAs/tsRNA-Val-AAC-017/Diagrams/tRF-Val-AAC-017_vs_FTMT22000006530(CATG00000077030.1).pdf]
